# New Insights into and Emerging Roles of Animal Models for Neurological Disorders

**DOI:** 10.3390/ijms23094957

**Published:** 2022-04-29

**Authors:** Changjong Moon

**Affiliations:** Department of Veterinary Anatomy and Animal Behavior, College of Veterinary Medicine and BK21 FOUR Program, Chonnam National University, Gwangju 61186, Korea; moonc@chonnam.ac.kr

Many researchers rely on animal studies to elucidate the mechanisms underlying diverse disease processes and to test the safety of emerging medical interventions. Animal research serves as a bridge between in vitro experiments and human trials in the preclinical stage. The majority of animal research employs almost genetically identical laboratory-bred mice or rats. Testing genetically similar individuals in a laboratory environment provides a level of control not available in clinical trials and is crucial for all phases of biomedical research. Numerous animal models have been developed to imitate pathophysiological abnormalities observed in humans. For an animal model of a human disease to be optimal, it must have similar pathophysiology, phenotypic, and histopathological traits; predictive biomarkers for disease progression or prognosis; therapeutic responsiveness; and levels of pharmacological safety or toxicity responses [1,2]. Thus, four types of animal models are employed in preclinical research: disease induction models, xenograft models, inbred strains, and transgenic models [2]. 

Various animal models have been critical in basic scientific and preclinical research on human neurological disorders, including traumatic or non-traumatic, neuroinflammatory, neuropsychological, and neurodegenerative diseases. The animal models are designed to mimic several facets of the disorders, including their genetic basis, neuropathological lesions, and clinical symptoms [3]. These animal models advance our understanding of the etiopathogenesis of neurological disorders, with the ultimate objective of formulating therapeutics that will eventually lead to the modification and/or prevention of neurological disorders. Despite the vast amount of previously acquired knowledge, researchers and the general public can benefit from novel animal models that more accurately mimic human diseases, including updates to previously established animal models and new insights into the molecular mechanisms underlying the disorders. However, positive perceptions toward animal modeling of neurological disorders are frequently tempered by difficulties in applying animal models to preclinical testing for potential therapeutic compounds [4]. 

Hence, the Special Issue “Modeling Neurological Disorders in Experimental Animals: New Insights and Emerging Roles [5]” aimed to present new interesting developments and offer innovative insights into the multifaceted pathophysiology of neurological disorders. Eminent researchers have proposed various animal models for studying molecular mechanisms underlying neurological disorders. This Special Issue contained nine original research articles on the differential gene expression analysis of genetic models, development of new models, further characterization of established models, and therapeutic studies utilizing animal models, in addition to eight review articles summarizing and highlighting recent advances in this field.

The research articles in this Special Issue have mostly focused on the alterations of behavioral and histopathological characteristics and signal transduction, as well as the testing of potential therapeutic compounds in genetically manipulated and/or exogenous factor-induced animal models for neurological disorders. To begin, the articles by Ang et al., entitled “Transcriptome Profiling Reveals Novel Candidate Genes Related to Hippocampal Dysfunction in SREBP-1c Knockout Mice” [6] and “SREBP-1c Deficiency Affects Hippocampal Micromorphometry and Hippocampus-Dependent Memory Ability in Mice” [7], are continuations of a previous study [8]. Sterol regulatory element-binding protein-1c (SREBP-1c) is a crucial modulatory molecule in lipid homeostasis. These studies established that SREBP-1c deficiency results in neurobehavioral abnormalities, including schizophrenia-like behaviors, and demonstrated that transcriptomes in the hippocampus of SREBP-1c knockout mice provided novel molecular evidence for the modulatory role of SREBP-1c in the mouse hippocampus and that the contributions of SREBP-1c to the structural plasticity of the mouse hippocampus may have contributed to the behavioral alterations. These novel findings provide insights into the important role of SREBP-1c in hippocampal functions.

This Special Issue contains two additional research articles on animal models of schizophrenia. Chang et al. reported in their article “Interaction of Prenatal and Postnatal Risk Factors in the Behavioral and Histological Features of a “Two-Hit” Non-Genetic Mouse Model of Schizophrenia” [9] that disruptions in brain development during the prenatal or postnatal period affect the structure and function of the brain, increasing susceptibility to mental disorders such as schizophrenia. Percelay et al. demonstrated in their article “Functional Dysregulations in CA1 Hippocampal Networks of a 3-Hit Mouse Model of Schizophrenia” [10] that this electrophysiological study highlighted dysregulations of functional properties and plasticity in hippocampal networks of three-hit mice, one of the mechanisms suspected to contribute to the pathophysiology of schizophrenia. These articles reinforce the face validity of non-genetic animal models that will help consider new therapeutic strategies for psychiatric disorders.

Another article in this Special Issue demonstrated a non-genetic and exogenous factor-induced animal model of neurological disorders. Although exposure to radiofrequency electromagnetic fields (RF-EMFs) has increased in the pediatric population, data on the consequences of the exposure to the central nervous system to RF-EMFs in them are scarce. The article by Kim et al. entitled “Exposure to RF-EMF Alters Postsynaptic Structure and Hinders Neurite Outgrowth in Developing Hippocampal Neurons of Early Postnatal Mice” [11] reveled that impaired neuronal outgrowth following RF-EMF exposure may result in a decrease in overall synaptic density during early neurite development of hippocampal neurons, as well as memory dysfunctions.

This Special Issue featured a research article testing a prospective therapeutic drug in a genetically manipulated mouse model of neurological disorder. Ding et al., in their article “Carbamazepine Restores Neuronal Signaling, Protein Synthesis, and Cognitive Function in a Mouse Model of Fragile X Syndrome” [12], uncovered that carbamazepine, an FDA-approved drug previously used to treat seizure and neuropathic pain, improves cognitive deficits, hyper-locomotion, and social deficits in Fragile X syndrome (FXS) mice, possibly via the downregulation of the elevated levels of ERK and Akt signaling, as well as protein synthesis in FXS mouse neurons. Conversely, it has been suggested that a genetical modification can alleviate neurological disorder symptoms. An article by Stetter et al. entitled “Amelioration of Cognitive and Behavioral Deficits after Traumatic Brain Injury in Coagulation Factor XII Deficient Mice” [13] has shown that FXII deficiency is associated with efficient post-traumatic behavioral and neuroendocrine recovery.

Additionally, in a research paper on the hormonal effect on maternal behavior, the article by Leko et al. entitled “Transcriptome Sequencing in the Preoptic Region of Rat Dams Reveals a Role of Androgen Receptor in the Control of Maternal Behavior” [14] revealed that androgen receptor (AR) levels are suppressed in the preoptic area of mothers, which is possibly mediated by an altered WD repeat domain containing 1 expression to allow sustained high-level care for the pups. Thus, they first implicated AR in the regulation of maternal behaviors. Although this article did not directly address neurological diseases, I included it in the invitation because investigating the possibility of hormone involvement in neurological dysfunctions using animal models can help advance novel therapeutic strategies for neurological disorders. 

Apart from the rodent models mentioned above, this Special Issue included an article describing the development of a zebrafish model that mimicked a human disease. Mucopolysaccharidosis IIIA (MPS IIIA, Sanfilippo syndrome type A), a pediatric neurological lysosomal storage disease, is caused by a deficiency of the enzyme N-sulfoglucosamine sulfohydrolase (sgsh), resulting in an impairment in heparan sulfate-glycosaminoglycan catabolism and its accumulation in tissues. The article “An Engineered *sgsh* Mutant Zebrafish Recapitulates Molecular and Behavioural Pathobiology of Sanfilippo Syndrome A/MPS IIIA” by Douek et al. [15] suggested that the *sgsh* (Deltaex5-6) zebrafish mutant could be a valuable resource for gaining a better understanding of MPS IIIA pathobiology and developing timely and effective therapeutic interventions.

The review articles presented in this Special Issue demonstrated the ingenuity of researchers in addressing the critical task of modeling human neurological disorders in animals in meaningful ways. The articles mainly reviewed various animal models for neurological disorders, including amyotrophic lateral sclerosis (ALS), Alzheimer’s disease (AD), epilepsy, Parkinson disease (PD), peripheral nerve injury, and post-traumatic stress disorder (PTSD). These animal models will be useful to gain better understanding of the mechanisms leading to signs of neurological disorders, validating drug targets, and providing assurance that therapeutic approaches will ultimately benefit patients. 

ALS is a fatal, multigenic, multifactorial, and non-cell autonomous neurodegenerative disease characterized by upper and lower motor neuronal loss. Bonifacino et al. [16] reviewed the most recent and accessible ALS genetic animal models, categorizing them according to the distinct genetic mutations and species and highlighting their modeling characteristics, the beginning and course of the disease, and their unique pathological hallmarks. Additionally, they emphasized the similarities and differences, as well as the advantages and disadvantages, with the goal of assisting researchers in selecting the most appropriate experimental animal model for a preclinical ALS investigation [16]. Liguori et al. [17] provided an updated and comprehensive review of how eukaryotic unicellular and multicellular organisms that replicate several of the main clinical features of the disease have aided in dissecting the pathological pathways of the disease insurgence and progression in ALS research.

AD is the most common cause of dementia, and its pathogenesis is complex, including Aβ deposits, tau aggregates, excitotoxicity, and neuroinflammation. Robert et al. [18] reviewed the recent studies on patient-derived tau seeding-based animal models and discussed their significance in enhancing our understanding of tau pathology and testing tau-based therapeutics. Additionally, current research on genetic epilepsy (GE) has discovered common mechanisms between GE and neurodegenerative diseases, including AD [19]. In GE, an increased epileptiform activity is almost certainly a primary pathology, contributing directly to cognitive impairment. Studies on GE could provide unique insights into AD pathogenesis. Thus, Kang [20] reviewed the epileptic mechanisms shared by AD.

PD is the second most prevalent neurodegenerative disorder after AD. No cure for PD has been discovered, and treatment strategies are aimed at relieving symptoms through the restoration of dopaminergic functions. Liu and Cheung [21] reviewed stem cell applications in the treatment of PD and how stem cell research has advanced our understanding of the disease, predicted the success of novel neuroprotective therapeutics, and identified crucial areas for future research.

Neurons are structurally distinct and have injury-prone dendrites and axons. Peripheral nerves that have been injured, but not central nerves, have the ability to recover and reinnervate their target organs. Numerous animal models have been used to elucidate the mechanisms of axon regeneration. Lee and Cho [22] reviewed the key experimental models that revealed the critical mechanisms regulating axon regeneration and degeneration in different systems and discussed the advantages of using rodent models when considering their application to understanding human diseases and for developing therapeutic methods. Gordon [23] reviewed (1) the processes of axon outgrowth from proximal to distal nerve stumps, (2) the growth of these axons within the stumps and intramuscular nerve pathways to target denervated muscle fibers, (3) the selection and number of muscle fibers reinnervated by the regenerated motor nerve fibers, and (4) the recovery of the properties of the nerve and muscle fibers after muscle reinnervation.

Ketamine has evolved into a versatile drug, which is used for a number of indications ranging from trauma analgesia to depression and PTSD therapy. Due to the inherent ethical problems associated with performing prospective clinical studies on traumatic events and stress-related diseases in humans, researchers frequently use preclinical rat models to investigate the effects of ketamine on fear memory and PTSD-like behaviors. A review by Choi et al. [24] summarized the current preclinical literature on ketamine and fear memory, focusing on the route, dosage, and timing of ketamine administration in rodent fear-conditioning studies. Additionally, the review discussed the molecular mechanisms through which ketamine exerts its influence on fear memory and stress-related behaviors [24].

Consequently, this Special Issue provides an update on current knowledge of the importance of animal models in mechanistic and therapeutic research on neurological disorders. I hope that readers will gain an understanding of current research trends and insights on future research directions through this Special Issue. However, I recognize that this Special Issue is insufficient to address the diverse animal models for a variety of neurologic disorders and that the articles included in this issue cannot possibly cover all on this topic. Thus, to complement any latest research data or research trends, I have re-launched the Special Issue “Modeling Neurologic Disorders in Experimental Animals: New Insights and Emerging Roles 2.0 [25]” to solicit new submissions of research and review papers. I expect that a large number of researchers with an interest in this topic will participate.
